# A retrospective study of irreversible electroporation for tumors adjacent to perihepatic important structure

**DOI:** 10.3389/fonc.2024.1387952

**Published:** 2024-09-12

**Authors:** Ju Gong, Shunhong Wang, Shuting Wang, Chaojie Li, Wenhua Li, Yingjie Chen, Ning Xia, Chen Wang, Zhongmin Wang

**Affiliations:** ^1^ Department of Interventional Radiology, Ruijin Hospital Luwan Branch, Shanghai JiaoTong University School of Medicine, Shanghai, China; ^2^ Department of Interventional Therapy, The Second Affiliated Hospital of Soochow University, Jiangsu, China; ^3^ Department of Intervention Vascular, The Third Affiliated Hospital of Shihezi University, Shihezi, China; ^4^ Ruijin Hospital, School of Medicine, Shanghai Jiao Tong University, Shanghai, China

**Keywords:** irreversible electroporation, ablation, interventional radiology, tumor, liver metastasis

## Abstract

**Background:**

Irreversible electroporation has been proved as a feasible and safe method against tumor in liver. However, few studies focused on tumors adjacent to perihepatic important structure like vessels, biliary system and gall bladder. These structures limit the effectiveness of conventional treatments. The aim of this article is to analyze the clinical outcomes of patients with hepatic tumors at the special sites who received IRE treatment and provide reliable evidence for broadening the scope of IRE’s clinical application.

**Methods:**

The clinical information of patients who underwent IRE ablation for tumors adjacent to perihepatic important structure between February 2017 and December 2021 was collected and retrospectively analyzed. All patients underwent contrast-enhanced CT or MRI for further evaluation at the 1-month follow-up and every 3 months thereafter. Post-ablation complications, recurrence, progression-free survival and overall survival were evaluated to analyze the prognosis of IRE ablation adjacent to perihepatic important structure. Categorical variables are presented as numbers followed by percentages. Continuous data are presented as the mean ± deviation. The tumor size and IRE ablation size were evaluated by the maximum diameters.

**Results:**

Thirty-two patients who underwent IRE ablation for tumor adjacent to perihepatic important structure were studied in this research. There were 39 lesions in 32 patients treated with IRE ablation. Fourteen of them (35.9%) were located adjacent to the porta hepatis, and 8 of them (20.5%) were located adjacent to the hepatocaval confluence. Subcapsular lesions accounted for 15.4% (6 of 39 lesions). The other 11 lesions were in the para gallbladder (5 of 39 lesions, 12.8%), the caudate lobe (5 of 39 lesions, 12.8%) and the colonic hepatic flexure (1 of 39 lesions, 2.6%). According to the Clavien−Dindo classification system for complications, all relative patients with cancer experienced complications below class III except one patient who developed postoperative hemorrhagic shock and improved after timely treatment. Recurrence *in situ* was observed in 5 of 32 (15.6%) patients. The median PFS of the patients who received IRE ablation was 384 days, and the median OS was 571 days.

**Conclusion:**

IRE ablation is a feasible and safe treatment strategy for tumors adjacent to perihepatic important structure. With improved equipment, optimized therapeutic parameters and long-term clinical trials, IRE will play an increasingly important role in the treatment of tumors in liver.

## Introduction

Liver cancer including primary hepatic tumor and liver metastasis is the fourth most deadly type of cancer worldwide that has an increasing rate of death and a consistently low survival rate (1). At present, in clinical practice, surgical resection is still considered the main treatment strategy for primary hepatic carcinoma. However, it was reported that only 10-15% of patients with liver metastases will experience long-term benefits of resection, and about 30% of the liver cancer patients are suitable for resection (2, 3).

Nowadays, minimally invasive techniques are rapidly developing, and interventional therapy, especially ablative treatment combined with chemotherapy, targeted therapy, or immunotherapy, is feasible and effective for patients with unresectable liver metastases or multiple liver tumors (4–6). Remarkably, ablative treatment for small liver tumors (maximum diameter is less than 3 cm) is expected to achieve comparable effects as surgical resection (7). However, ablative therapy is ineffective in treating some liver tumors at special sites. For example, ablative therapy for residual lesions adjacent to the porta is most often ineffective due to the heat sink effect and the safe distance between the needle electrode and visceral vessels, and tumors near the biliary system and hepatic flexure have an increased risk of iatrogenic perforations after ablation and tumors adjacent to the diaphragm, especially those protruding beyond the outline of the liver, are prone to diaphragmatic injury and diaphragmatic hernia because of ablation (8–10).

Irreversible electroporation (IRE), a relatively new modality to treat cancer, uses high-voltage electrical pulses to induce cancer cell apoptosis through nanoscale irreversible cell membrane pores, which results in an imbalance of ion exchange and destruction of the homeostasis of the intracellular environment (10, 11). The main advantage of IRE is that there is no significant temperature change during the process and no severely irreversible damage to adjacent normal tissue (12). And IRE is not affected by the heat sink effect. This kind of highly selective ablation modality is especially suitable for hepatic carcinoma adjacent to perihepatic important structure. In several studies, researchers have reported the feasibility and safety of IRE in the liver, however few studies show a focus on tumors adjacent to perihepatic important structure like porta hepatis, hepatocaval confluence, subcapsular, gall bladder, caudate lobe and hepatic flexure of colon (13, 14). Therefore, the treatment strategies for these types of tumors are supposed to be discussed in clinical practice. The aim of this research is to analyze the clinical outcomes of patients with hepatic tumors adjacent to perihepatic important structure who receive IRE treatment and provide reliable evidence for broadening the scope of clinical application of IRE.

## Materials and methods

**Figure 1:**
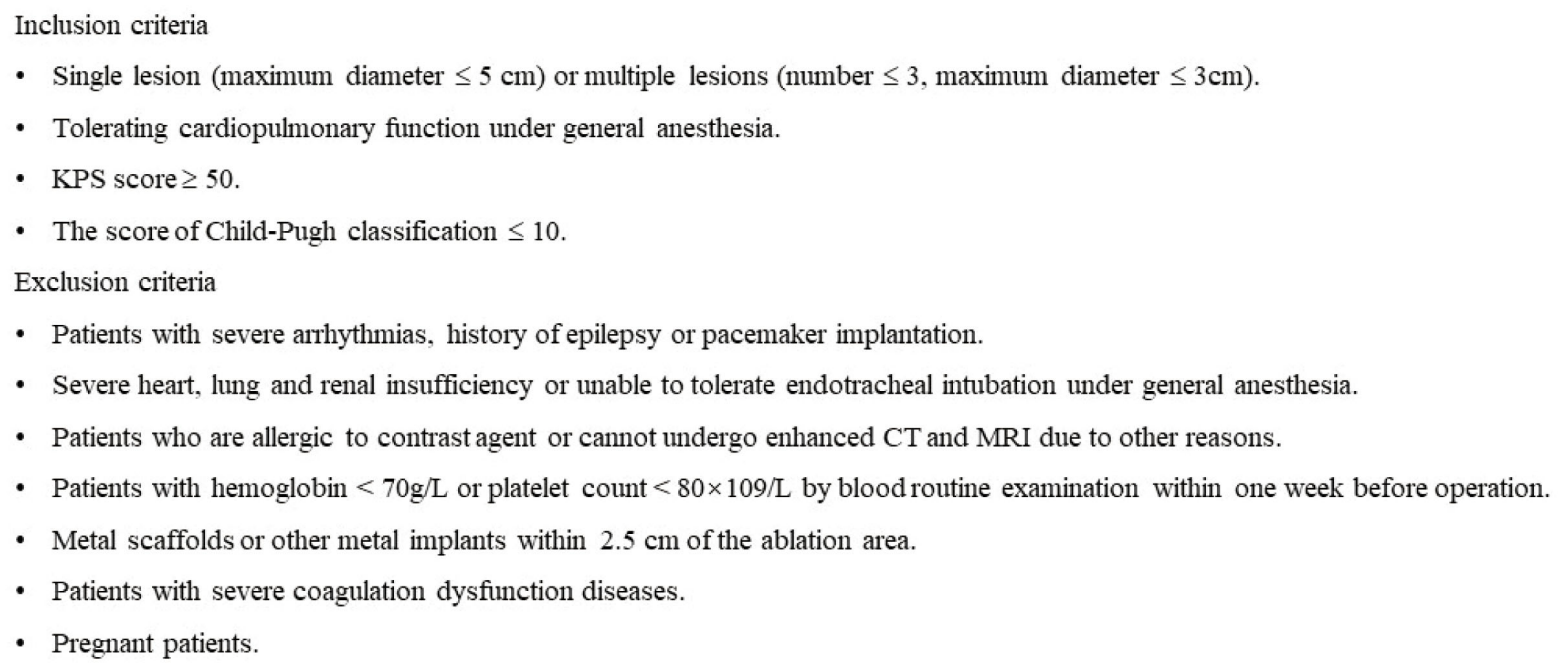
The inclusion and exclusion criteria of IRE ablation for tumors in special sites of liver.

This retrospective study was approved by the institutional review board. All patients with liver tumors were assessed preoperatively using the following criteria in **Figure 1** to determine suitability for IRE treatment. However, only patients who underwent IRE for tumors adjacent to special sites, including the diaphragm, capsule, inferior vena cava, hepatic vein, gall bladder or secondary branch of the portal vein, were included in this study. All patients signed informed consent forms.

### Equipment

A NanoKnife (Angiodynamic, USA) was used for IRE treatment. The main needle electrode was a 19G monopolar needle electrode, which was combined with other electrodes to adapt to the size and shape of tumors. A total of 2 to 5 electrodes were used in this research according to the parameter of tumors and IRE settings. There is an insulation layer around the needle electrode. The exposed section of the electrode can be adjusted by adjusting the length of the insulation layer subcutaneously. The common electrode exposure length is 1-2cm.

### Procedure

#### Preoperative preparation

An anesthetist, an itinerant nurse, and an experienced equipment technician comprised the IRE treatment team to assist the interventional radiologists. General anesthesia combined with neuromuscular blockade was required to reduce the effects of muscle contractions.

#### The image-guiding mode of IRE

Multiplanar reconstruction using CT showed the relationship between the electrode and lesions or the perifocal structures satisfactorily and the electrode distribution could be adjusted in real time.

#### The setting of electrode route (pre-operation plan)

Contrast-enhanced CT and multiplanar reconstruction were performed to obtain images to determine the location and size of the lesions and to show the relationship between the lesions and the adjacent structures. Then, the access and amount of needle electrodes were chosen, and interventional radiologists placed the electrodes in the planned positions. The electrodes were supposed to be parallel to each other, and the distance between every two electrodes was 1 to 2.2 cm. The ideal electrode route form an ablation area that covers the whole tumor in all cases.

#### The strategy for IRE treatment

The parameters of the NanoKnife system were adjusted after confirming suitable electrode positions. The IRE planning system was used to adjust the appropriate ablation parameters to achieve the ablation area covering all tumors. Initially, test pulses (20 pulses of 1500 V/cm for 70 μs) were delivered to evaluate the impedance and current of the tissue. Then the IRE ablation parameters were set as follows: voltage 1500V/cm, 100 pulses/2 cycles, pulse width 70-90μs. electrode distribution was adjusted for an ideal ablation zone, including the margins of the target lesion and the surrounding normal tissue. Meanwhile, the variation trend of current (2550-3000mA) should be closely monitored during the operation. For some cases, electrodes were repositioned between applications of pulses according to the monitored images.

#### Assessment of the IRE procedure and follow-up

**Figure 2:**
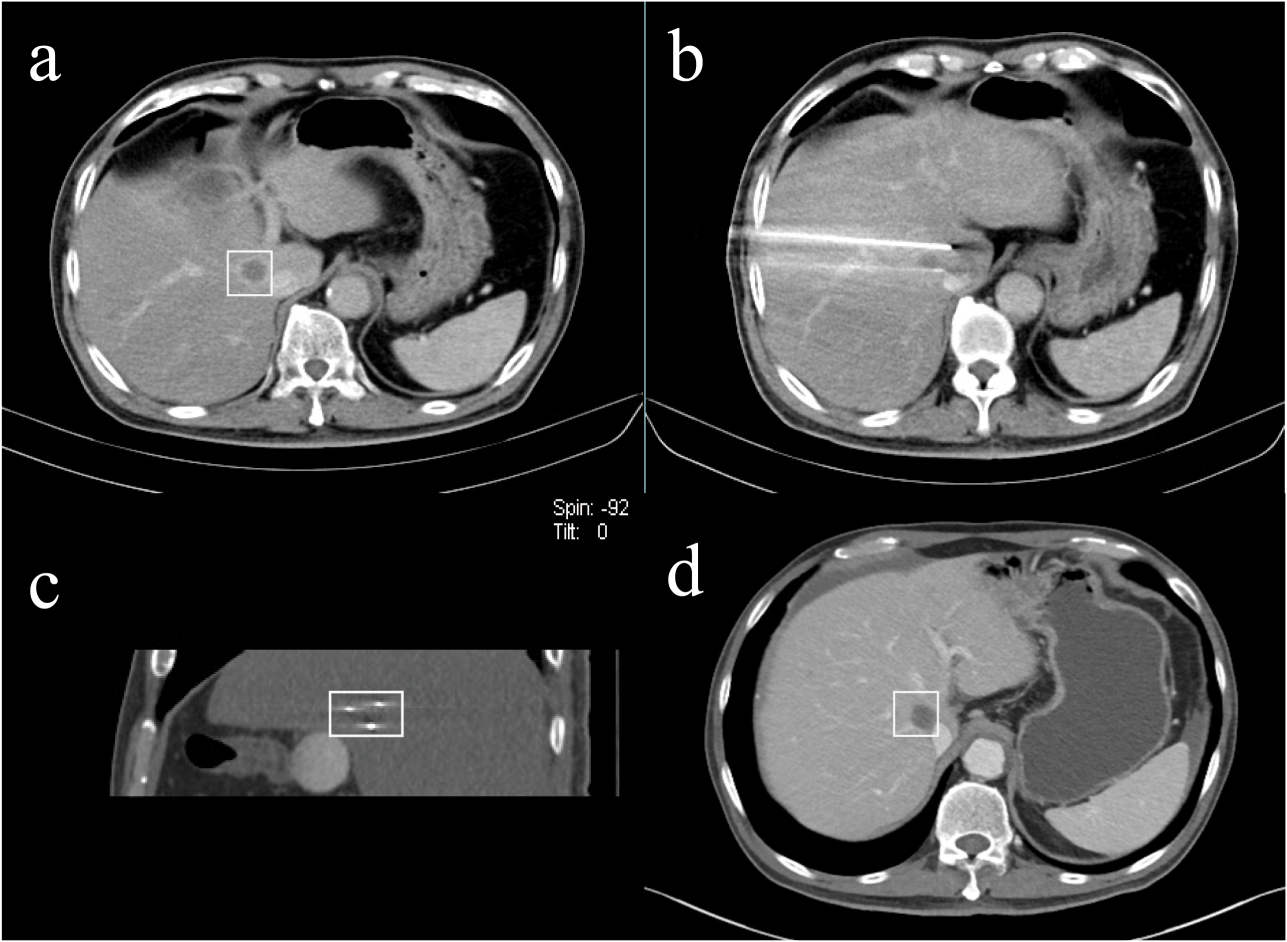
A 66-year-old male with liver metastasis of pancreatic cancer adjacent to the superior vena cava underwent IRE ablation. [**(A)** venous phase of preoperative enhanced CT (rectangular); **(B, C)** the approximate equilateral triangle distribution of electrodes; **(D)** venous phase of postoperative enhanced CT at the 13th-month follow-up indicated satisfactory ablation effect (rectangular)].

**Figure 3:**
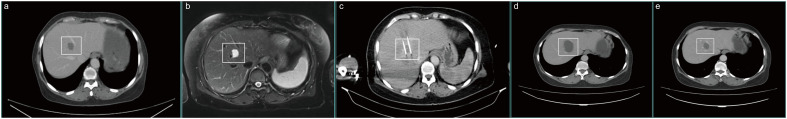
A 54-year-old female patient with liver metastasis of colon cancer underwent IRE ablation for the lesion adjacent to middle hepatic vein. [**(A, B)** preoperative enhanced CT and MRI; **(C)** the distribution of electrodes; **(D)** postoperative CT at the 3-month follow-up; **(E)** postoperative CT at the 19-month follow-up].

Contrast-enhanced CT was performed immediately to assess the completeness of IRE ablation and to detect intraoperative complications. IRE ablation was considered to be successful if there was no obvious enhancement in the arterial phase or washout in the venous phase or the delayed phase. All patients receiving IRE underwent contrast-enhanced CT or MRI for further evaluation at the 1-month follow-up and every 3 months thereafter (**Figures 2**, **3**). Recurrence was defined as the appearance of new lesions on contrast-enhanced CT or MRI at the follow-up. Lesions adjacent to the primary IRE site were defined as recurrence *in situ*, while those away from the IRE site were termed recurrence *ex situ*. Postablation complications were classified into five grades according to the Clavien−Dindo classification (15). The follow-up treatment received by the patients should be recorded during follow-up. Progression-free survival (PFS) was defined as the time interval from IRE ablation to tumor progression or death. Overall survival (OS) was defined as the time interval from IRE ablation to death from any cause (for patients who were lost to follow-up before death, the time of death was counted as the last follow-up).

#### Statistical analysis

Categorical variables are presented as numbers followed by percentages. Continuous data are presented as the mean ± deviation. The tumor size and IRE ablation size were evaluated by the maximum diameters. PFS and OS were calculated by using GraphPad Prism 8 (GraphPad Software).

## Results

### Patient characteristics

Thirty-two patients (22 male, 10 female) who underwent IRE ablation between February 2017 and December 2021 were included in this study (**Table 1**). Regarding underlying health problems, hypertension was diagnosed in 7 (21.9%) patients, diabetes mellitus in 6 (18.8%) patients, and heart disease in 3 (9.4%) patients. Furthermore, 6 of 32 patients (18.8%) had a confirmed history of hepatitis or cirrhosis.

**Table 1:** Characteristics of patients.

Variable	Data
Total number of patients	32
Sex	
Male Female	22 (68.7%) 10 (31.3%)
Average age	60.59 ± 9.52 years
Hypertension	7 (21.9%)
Diabetes mellitus	6 (18.8%)
Heart disease	3 (9.4%)
KPS value	
50-80 (including 80) 80-100	12 (37.5%) 20 (62.5%)
Child-Pugh class	
A B C	22 (68.7%) 7 (21.9%) 3 (9.4%)

The Karnofky Performance Status (KPS) of the patients was evaluated before IRE ablation. All patients received a score of more than 50. Among them, 20 patients (62.5%) were in the independent state, and the other 12 patients (37.5%) were in the semi-independent state. For the Child−Pugh class of liver function, 22 of 32 patients (68.7%) were divided into Class A, 7 of 32 patients (21.9%) were divided into Class B, and the other 3 patients (9.4%) were divided into Class C, but all of them received a score of 10, which indicated that they were in a relatively tolerating state.

### Tumor characteristics

All the primary tumors of 32 patients were confirmed pathologically by comprehensive analysis of tissue biopsy, clinical findings and imaging characteristics (**Table 2**). Ten of 32 (31.3%) patients were suffering from pancreatic carcinoma as the primary tumor, and 7 of 32 (21.9%) patients were diagnosed with hepatic carcinoma. Other primary tumor types in this study are shown in **Table 3**. There were 39 lesions in 32 patients treated with IRE ablation. Fourteen of them (35.9%) were located adjacent to the porta hepatis, and 8 of them (20.5%) were located adjacent to the hepatocaval confluence. Subcapsular lesions accounted for 15.4% (6 of 39 lesions). The other 11 lesions were in the para gallbladder (5 of 39 lesions, 12.8%), the caudate lobe (5 of 39 lesions, 12.8%) and the colonic hepatic flexure (1 of 39 lesions, 2.6%). The maximum diameter of 15 (38.5%) lesions ranged between 2 cm and 3 cm and that of 13 (33.3%) lesions ranged between 3 cm and 4 cm. There were also 8 (20.5%) lesions with a maximum diameter of less than 2 cm and 3 (7.7%) lesions with a maximum diameter of more than 4 cm.

**Table 2:** Characteristics of tumors.

Variable	Data
Primary tumor types	
Pancreatic carcinoma Hepatic carcinoma Intestinal carcinoma Cholangiocarcinoma Gastric carcinoma Nasopharyngeal carcinoma Breast carcinoma	10 (31.3%) 7 (21.9%) 6 (18.8%) 5 (15.6%) 2 (6.2%) 1 (3.1%) 1 (3.1%)
Total number of lesions treated with IRE	39
The tumor sites treated with IRE	
Porta hepatis Hepatocaval confluence Subcapsular Gall bladder Caudate lobe Hepatic flexure of colon	14 (35.9%) 8 (20.5%) 6 (15.4%) 5 (12.8%) 5 (12.8%) 1 (2.6%)
Tumor size	
<2 cm 2-3 cm 3-4 cm >4 cm	8 (20.5%) 15 (38.5%) 13 (33.3%) 3 (7.7%)

**Table 3:** Characteristics of IRE ablation.

Variable	Data
Number of electrodes	
2 3 4 5	15 (46.9%) 4 (12.5%) 12 (37.5%) 1 (3.1%)
Number of lesions per procedure	
1 2	25 (78.1%) 7 (17.9%)
Repeated number of IRE ablation per procedure	
2 4 6	12 (37.5%) 15 (46.9%) 5 (15.6%)
Electrode spacing, average	1.89 ± 0.43 cm
Exposure length of electrodes, average	2.12 ± 0.27 cm
IRE ablation size	
<3 cm 3-4 cm 4-5 cm >5 cm	5 (12.8%) 17 (43.6%) 12 (30.8%) 5 (12.8%)
Average IRE area of cross section	15.91cm^2^

### IRE characteristics

The number of electrodes, lesions per procedure and repeated IRE ablations per procedure were determined by the IRE team according to the intraoperative positioning and real-time adjustment. Related data are described in **Table 3**. The average effective ablation distance between the electrode needles was 1.8 ±0.43 cm, and the electrode needles were parallel to each other. The electrode needles were arranged to ensure that the entire ablation area covered all the lesions and extended beyond the edge by 1 cm. The average exposure length of the electrodes was 2.1 ± 0.27 cm, which was adjusted according to the location of the lesions. The zone of IRE ablation was evaluated immediately by abdominal contrast-enhanced CT or MRI in the postablation imaging assessment. Most of the imaging showed a reduced density of the liver tissue in the ablation area, and the degree of enhancement was lower than that of the surrounding liver tissue. There were also bubble shadows of different sizes in the ablation area. The maximum diameter of the IRE ablation zone was 3-4 cm in 17 of 39 lesions (43.6%), 4-5 cm in 12 of 39 lesions (30.8%), and less than 3 cm or more than 5 cm in 10 of 39 lesions (25.6%). The average IRE area of cross section was 15.91cm^2^.

### Outcomes

**Figure 4:**
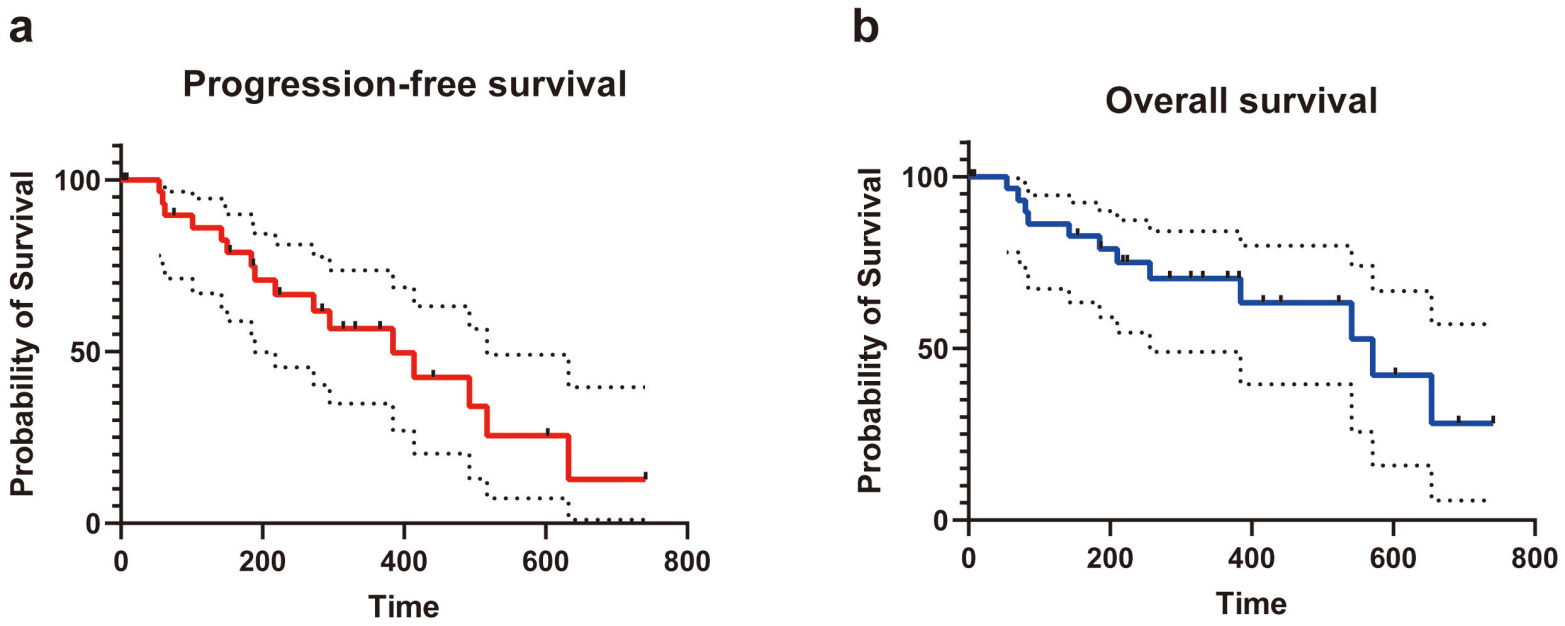
The progression-free survival **(A)** and overall survival **(B)** of patients underwent IRE for tumors in special sites of liver.

According to the Clavien−Dindo classification for complications, 7 of 32 (21.9%) patients developed class I complications. Five of 32 (15.6%) patients developed class II complications, and 4 of 32 (12.5%) patients suffered from class III complications (drainage for pleural or peritoneal effusions). One patient had a class IV complication (hemorrhagic shock). The most common complication was pleural or peritoneal effusions (8 of 32 patients, 25.0%). One patient was transfused with 2 units of platelets because of the critical value after IRE ablation. Another patient developed postoperative hemorrhagic shock and improved after timely treatment. Regarding the subsequent treatment after IRE ablation, 6 of 32 (18.8%) patients received systemic chemotherapy, 3 of 32 (9.4%) patients received transcatheter arterial infusion, and 2 of 32 (6.3%) patients received immunotherapy. Recurrence *in situ* was observed in 5 of 32 (15.6%) patients. The median overall PFS of the patients who received IRE ablation was 384 (95%CI: 361-403) days, and the median OS was 571 (95%CI: 545-595) days (**Figure 4** and **Table 4**).

**Table 4:** Outcomes of patients underwent IRE ablation.

Variable	Data
Post-ablation complications	
I II III IV V	7 (21.9%) 5 (15.6%) 4 (12.5%) 1 (3.1%) 0
Post-ablation treatment	
Systemic chemotherapy Arterial infusion Immunotherapy	6 (18.8%) 3 (9.4%) 2 (6.3%)
Recurrence in situ	5 (15.6%)
Median progression-free survival	384 d
Median overall survival	571 d

## Discussion

Thermal ablation techniques (including radiofrequency ablation and microwave ablation) have been widely used in the radical treatment of tumors in liver and have achieved curative effects similar to those of surgical treatment for small liver cancer. It is advantageous because it is a simple operation, causes minimal trauma, has a quick recovery and a short hospitalization period. However, thermal ablation still has certain limitations. On the one hand, when the lesion is adjacent to a large blood vessel, the temperature of the targeted region is likely to be lower than 60°C due to the “heat sink effect” of blood flow, which has a major influence on the effect of thermal ablation. On the other hand, high temperature may damage the large blood vessels, bile duct, gallbladder and gastrointestinal tract, which could cause complications such as bleeding, liver abscess, gallbladder perforation and gastrointestinal perforation (16, 17). However, some evidence of thermal ablation with hydrodissection technique has confirmed safe for perivascular, peripheral and subdiaphragmatic tumors (18, 19). Some techniques using hypothermia induction chemotherapy have also been reported (20).

According to its special mechanism, irreversible electroporation therapy, in theory, only kills cells with lipid bilayers and has little effect on the fibrous structure and collagen tissue in vessels and organ lumen (21). Cells in blood vessels, bile ducts, gallbladder and gastrointestinal tract adjacent to the liver tissue were induced to undergo apoptosis during irreversible electroporation. However, normal cells can regenerate and repair in a relatively short time because the frame structure of the organ still exists (22). Therefore, the important structure will not be affected. Moreover, because apoptotic vascular endothelial cells shed within 7 days after IRE ablation, an embolus may form in small blood vessels and further reduce the risk of bleeding (23, 24).

In this study, 39 lesions in 32 patients underwent IRE ablation. Although these lesions were in special locations, the technical success rate was still 100% without severe intraoperative complications. Safety is particularly important for the clinical application of IRE ablation. It’s worth noting that circumferential enhancement bands related to hyperemia may be seen in the surrounding liver tissue during the venous phase but should not be considered active lesions. The common complications that have been reported include liver abscess, hemorrhage, pneumothorax, atrial fibrillation, and pleural effusion (25–27). In this research, postoperative complications were divided into five grades according to the Clavien−Dindo classification for complications. The postoperative complication with the highest incidence in this study was pleural or peritoneal effusion. In this study, the reason for the high risk of pleural or peritoneal effusion is thought to be the special locations of the lesions. These special locations are often close to the capsule, and inflammatory stimulation occurs during IRE ablation. However, some peritoneal effusions are more likely bloody or bilious. It should be noticed for the possibility of hemorrhage or biliary fistula. In this study, one patient developed hemorrhagic shock, which improved after timely treatment. For all types of ablations, hemorrhage is often a common complication, and damage to large vessels during ablation can sometimes be life-threatening. Similar to IRE, common complications after RFA include pleural effusion, liver function impairment, pneumothorax, hemoperitoneum. Skin burn and secondary biliary dilatation have also been reported (28, 29). Since IRE ablation does not have a thermal effect, the method of slow withdrawal is recommended, which uses self-blood clots to reduce the risk of bleeding. Moreover, gelatin sponges can be used to treat definite hemorrhage.

In our research, the average PFS of the patients who received IRE ablation was 315 days, and the median OS was 571 days. Residual tumor recurrence is a significant concern after ablation and surgery. Five of 32 (15.6%) patients experienced recurrence *in situ*, and 3 of 32 (9.4%) patients experienced recurrence *ex situ*.

There are some limitations in our research. First, the sample size was small, and there was a large difference in the number of cases between different locations. Second, there was a short follow-up period for some patients. Third, this study was retrospective, which may have caused selection bias.

## Conclusion

In conclusion, this study showed that IRE ablation is feasible and safe for lesions in special locations of the liver. With improved equipment, optimized therapeutic parameters and long-term clinical trials, IRE will play an increasingly important role in the treatment of tumors in liver.

The importance and relevance of this study to the field lie in several areas. Firstly, the study provides important insights into the safety and efficacy of IRE ablation for treating liver lesions adjacent to perihepatic important structure, which can inform clinical practice and help doctors make more informed treatment decisions. Secondly, the study highlights the need for further research and improvement in the treatment of liver tumors adjacent to perihepatic important structure, particularly in terms of reducing residual tumor recurrence rates.

In addition, the study’s findings underscore the importance of continued technological advancements and refinement of therapeutic parameters in the field of IRE ablation focusing on the tumors adjacent to perihepatic important structure. By addressing the limitations of this study, such as small sample size and short follow-up period, future research could further establish the role of IRE ablation in tumor treatment and potentially improve these patient’s outcomes. Overall, this study contributes valuable knowledge to the field and underscores the importance of continued research in this area.

## Data Availability

The original contributions presented in the study are included in the article/supplementary material. Further inquiries can be directed to the corresponding author.
